# Glycine-to-aspartic acid mutation at codon 51 in *Snca* disrupts the synaptic localisation of α-synuclein and enhances its propensity for synucleinopathy

**DOI:** 10.1093/braincomms/fcaf224

**Published:** 2025-06-06

**Authors:** Stephen West, Ammar Natalwala, Karamjit Singh Dolt, Douglas J Lamont, Melanie McMillan, Kelvin Luk, Tomoji Mashimo, Tilo Kunath

**Affiliations:** Centre for Regenerative Medicine, Institute for Regeneration and Repair, The University of Edinburgh, Edinburgh EH16 4UU, UK; Centre for Regenerative Medicine, Institute for Regeneration and Repair, The University of Edinburgh, Edinburgh EH16 4UU, UK; Department of Neuromuscular Diseases, UCL Queen Square Institute of Neurology, Queen Square House, London WC1N 3BG, UK; Victor Horsley Department of Neurosurgery, National Hospital for Neurology and Neurosurgery, Queen Square, London WC1N 3BG, UK; Centre for Regenerative Medicine, Institute for Regeneration and Repair, The University of Edinburgh, Edinburgh EH16 4UU, UK; FingerPrints Proteomics Facility, School of Life Sciences, University of Dundee, Dundee DD1 5EH, UK; Centre for Reproductive Health, Institute for Regeneration and Repair, The University of Edinburgh, Edinburgh EH16 4UU, UK; Department of Pathology and Laboratory Medicine, Center for Neurodegenerative Disease Research, Alzheimer's Disease Core Center, Institute on Aging, University of Pennsylvania Perelman School of Medicine, Philadelphia, PA 19104-2676, USA; Division of Animal Genetics, Laboratory Animal Research Center, Institute of Medical Science, The University of Tokyo, Minato-ku, Tokyo 108-8639, Japan; Centre for Regenerative Medicine, Institute for Regeneration and Repair, The University of Edinburgh, Edinburgh EH16 4UU, UK; School of Biological Sciences, Institute for Stem Cell Research, The University of Edinburgh, Edinburgh EH16 4UU, UK; Centre for Engineering Biology, The University of Edinburgh, Edinburgh EH9 3BF, UK

**Keywords:** α-synucleinopathies, G51D mutation, lewy pathology, proteomics, synaptic dysfunction

## Abstract

Point mutations in the *SNCA* gene, which encodes α-synuclein (αSyn), are a known cause of familial Parkinson’s disease. The glycine-51-aspartic acid (G51D) mutation causes early-onset neurodegeneration with complex, wide-spread αSyn pathology. We used CRISPR/Cas9 gene editing to introduce the G51D point mutation into the endogenous rat *Snca* gene. Our goal was to investigate whether the G51D αSyn mutation gives rise to synucleinopathy and neurodegenerative phenotypes in rats. Co-localisation immunostaining studies with synaptic proteins revealed that αSyn^G51D^ protein fails to efficiently localise to synapses. Furthermore, biochemical isolation of synaptosomes from rat cortex demonstrated a significant depletion of αSyn in *Snca^G51D/+^* and *Snca^G51D/G51D^* rats. Unbiased proteomic investigation of the cortex identified significant synaptic dysregulation in *Snca^G51D/G51D^* animals. Finally, we compared the propensity for synucleinopathy of *Snca^+/+^* and *Snca^G51D/G51D^* rats by stereotaxically delivering αSyn pre-formed fibrils (PFFs) into the pre-frontal cortex. At an early time-point, 6 weeks post-injection, we observed discrete Lewy pathology-like structures positive for phosphoserine-129-αSyn (pS129-αSyn) only in *Snca^G51D/G51D^* brains. At 26 weeks post-injection of PFFs, *Snca^G51D/G51D^* brains exhibited intense, discrete pS129-αSyn-positive structures, while *Snca^+/+^* brains exhibited diffuse pS129-αSyn immunostaining. In summary, G51D mutagenesis of the endogenous *Snca* rat gene caused reduced synaptic localisation of αSyn, proteomic evidence of early synaptic dysfunction, and enhanced propensity for αSyn pathology.

## Teaser

West and Natalwala *et al.* report on the first rat model with a Parkinson’s α-synuclein (αSyn) mutation. A single amino acid change of glycine-to-aspartic acid at codon 51 in the rat *Snca* gene results in a reduced localisation of αSyn to synapses and an increased propensity to acquire Lewy pathology.

## Graphical Abstract

**Graphical Abstract:**
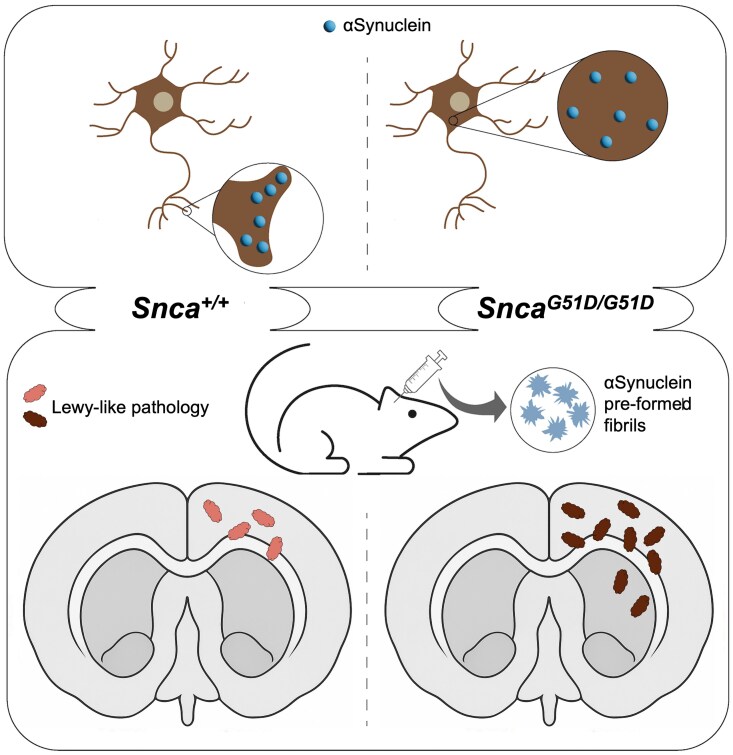

## Introduction

Most Parkinson’s disease cases are sporadic, and a relatively small number of cases are caused by *SNCA* multiplication or point mutations.^1,2^ The latter include the A30G, A30P, E46K, G51D, A53E, A53G, A53T, A53V and E83Q mutations.^3-9^ The G51D point mutation, in exon 3 of *SNCA*, was discovered in 2013, and reported in a French, British and later a Japanese family.^10-12^ The clinical manifestation of this mutation was that of early-onset and combined features of Parkinson’s disease, dementia with Lewy bodies, and multiple system atrophy, with clinical severity comparable to patients with a *SNCA* triplication mutation.^1^ Furthermore, glial cell inclusions were observed in G51D patient brains on autopsy.^10^ The frontal cortex, hippocampus and posterior striatum were sites of observed pathology, in addition to the substantia nigra.^13^ The accelerated disease progression in G51D patients makes this an important mutation to study and to delineate the underlying pathological mechanisms of conditions driven by α-synucleinopathy.

Limited animal models incorporating the G51D mutation have been generated so far, and whilst data suggests that this mutation leads to dopaminergic cell loss and diminished survival in *Drosophila*,^14^ this was not the case in a pig model (E46K, H50Q and G51D mutations combined).^15^ We generated an αSyn^G51D^ rat model using a CRISPR/Cas9 approach.^16^ We made a single amino acid mutation (glycine-51 to aspartic acid) to the endogenous rat *Snca* gene. This overcomes limitations associated with transgenic overexpression, such as unknown transgene integration sites, variable transgene expression and pleiotropic effects.^17^ We recently reported positron emission tomography data for our G51D rat model, showing an increase in dopamine turnover in the striatum in aged *Snca^G51D/G51D^* rats.^16^ However, there was no reduction in dopamine production. Since increased dopamine turnover prior to reduction of dopamine production is a sign of prodromal and early Parkinson’s in patients,^18-20^ we focussed our investigation on early molecular signs of synucleinopathy. It has been described that synaptic deficiencies and axonal die back occur well before overt dopaminergic neuron loss in PD,^21-23^ and that increased dopamine turnover in early PD is likely due to compensatory changes in pre-synaptic dopaminergic termini.^20^ Based on the biophysical properties of αSyn^G51D^  *in vitro* and its reluctance to form α-helices in the presence of lipids,^24^ we hypothesised αSyn^G51D^ would be less membrane bound *in vivo* and exhibit reduced localisation to synapses. Furthermore, when αSyn is in its α-helical form its ability to form fibrils is significantly reduced.^25^ This suggests that αSyn^G51D^, with its reduced ability to adopt α-helical conformations, will have an increased propensity to form fibrillar structures *in vivo*.

The overall aim of this study was to characterise the molecular and cellular phenotypes of the αSyn^G51D^ rat model. Based on the increased dopamine turnover we previously observed in this rat model,^16^ we hypothesised the integrity of synapses will be affected without overt loss of dopaminergic neurons. We directly investigated the synaptic localisation of αSyn^G51D^ protein by immunofluorescence confocal microscopy and co-localisation analysis with known synaptic proteins. We further analysed synaptosome preparations by an unbiased quantitative mass spectrometry approach and targeted western blotting. We demonstrate that unlike wild-type αSyn protein, αSyn^G51D^ is not significantly localised to synapses. We also investigated propensity for formation of Lewy pathology (LP)-like structures in the αSyn^G51D^ rat model since the ability of αSyn to form α-helices is inversely correlated to its ability to form fibrils.^25^  *Snca^G51D/G51D^* and *Snca^+/+^* rats were administered wild-type αSyn pre-formed fibrils (PFFs) by stereotaxic injection. We show that mutant animals have an increased propensity to synucleinopathy since they exhibited an accelerated kinetics of pathology formation *in vivo*.

## Materials and methods

### Animals

All animal experiments were conducted under the Project Licence No. PC6C08D7D in accordance with Home Office regulations under the Animals Scientific Procedures Act 1986. Rats used in this study were maintained on a Fischer 344 background and were either wild-type at the *Snca* locus or were heterozygous or homozygous for the *Snca^G51D^* mutant allele.^16^ Rats used in this study were given *ad libitum* access to food and water and were maintained on a 12-h light-dark cycle. All rats used were culled by schedule 1 CO_2_ inhalation followed by decapitation.

### Genotyping

Genotyping was performed as previously described.^16^ Briefly, genomic DNA was extracted from ear notches and *Snca* exon 3 was PCR amplified (forward primer 5′-TGGTGGCTGTTTGTCTTCTG-3′ and reverse primer 5′-TCCTCTGAAGACAATGGCTTTT-3′). The GGA to GAT (G51D) mutation created a new *Bsp*HI site in exon 3. PCR products were subjected to *Bsp*HI restriction enzyme digestion and agarose gel electrophoresis to distinguish wild-type, *Snca^G51D/+^* and *Snca^G51D/G51D^* mutant rats.

### Tissue dissection

Following schedule 1 cull, brains were removed, and depending on the downstream use, the brain was prepared using the following techniques:

1. Brain regions were sub-dissected and frozen on dry ice for synpatosome isolation, RNA isolation (for RT-qPCR), αSyn ELISA, or protein isolation for western blotting.
2. Brain was submerged in 4% PFA/PBS for 24 h followed by further processing for immunohistochemical analysis.

### RNA isolation

A pestle and mortar were cooled in a dry ice-ethanol bath and used to crush tissue to powder. RNA was extracted from ∼10 mg of tissue per animal. RNA was extracted using RNeasy Lipid kit (Qiagen, 74804), following manufacturer’s instructions. RNA was quantified using the Qubit 2.0 (ThermoFisher) system following manufacturer’s instructions. RNA quality was assessed using the TapeStation2200.

### RT-qPCR

RNase-free water (Ambion, AM9937) was added to the RNA (200 ng—1 μg) to give a 11 μL sample. The samples were incubated with 1 μL dNTP mix (10 mM, Life Technologies, 10297018) and 1 μL random primers (50 ng/μL, Thermo Fisher, PCR-545- 020T) at 65**°**C for 5 min and then chilled on ice. After brief centrifugation, 4 μL 5× SSIV Buffer (ThermoFisher, 18090010), 1 μL 0.1 M DTT (Life Technologies, Y00147) and 1 μL RNAse OUT (40 units/μL, Life Technologies, 10777019) were added. Then 1 μL Superscript IV reverse transcriptase (ThermoFisher, 18090010) or 1 μL RNase free water for the negative reverse transcriptase sample was added. This was mixed and incubated at room temperature for 10 min, followed by 10 min at 53**°**C. The reaction was inactivated by incubation at 80**°**C for 10 min. The cDNA mix was placed on ice and 60–180 μL RNase free water was added. The cDNA samples were then ready for qPCR.

qPCR was performed using the Roche LightCycler® 480 System with the Universal Probe Library (UPL) (Roche). The Roche UPL Assay design centre was used to design intron-spanning primers with a specific UPL probe (Table 1). Reactions containing primers, UPL Probe, UPL Probe Master Mix (Roche) and PCR water were performed in 386-well plates as described in the manufacturer’s instructions. The results were normalised to the geometric mean of two house-keeping genes (*Tbp*, *Hprt*) and then expressed relative to control samples.

**Table 1:** Primers and probes used in UPL qPCR system

Gene	Forward primer	Reverse primer	Probe
*Hprt*	ctcctcagaccgcttttcc	tcataacctggttcatcatcactaa	95
*Snca*	gcagtgaggcttatgaaatgc	aggcttcaggctcatagtcttg	6
*Tbp*	cccaccagcagttcagtagc	caattctgggtttgatcattctg	129

### αsyn ELISA

ELISA for αSyn protein (Bioassay Technology Laboratory, E1113Ra) was performed as manufacturer’s instructions. Optical density (OD) was determined in FLUOstar Omega (BMG LABTECH) plate reader set to 450 nm. Line of best fit is fitted to standards, and equation of the line is generated. This equation is used to calculate protein concentration of the samples from their OD.

### DAB immunohistochemistry

Following 24-h PFA fixation, tissue was washed 3×PBS (Gibco, 18912014) over 24 h. At this point tissue was either bisected and prepared for sagittal sections (αSyn analysis) or sub-dissected into regions of interest using a rat brain matrix (TH analysis). Regions were chosen based on the Rat Brain Atlas.^26^ The most anterior point of the cortex (bregma +5 mm) was used as a zero point. For striatal analysis 3 mm to 6.5 mm was dissected (bregma +2 to −1.5 mm) and for nigral analysis 9 mm to 12.5 mm (bregma −4.5 mm to −7.5 mm) was dissected. Dissected regions were then cryoprotected in 30% sucrose (SLS, CHE3650)/PBS (Gibco, 18912014) until the tissue sunk (usually 24–48 h) at 4°C. Tissue was then frozen on a metal chuck with optimum cutting temperature (OCT, CellPath, KMA010000A) compound in a dry ice ethanol bath and stored at −20°C until sectioning. Every 6th 30 um serial section was taken using the Leica cryostat (Cyrostar NX50) set at −20°C onto Superfrost slides (ThermoFisher, J1800AMNT).

Following sectioning slides were washed in TBS, and then blocked in 10% goat serum (GS, Sigma, G9023)/TBS with 0.1% Triton (Fisher, BP151–100) (10% GS/TBT) for 1 h. Slides were then incubated overnight in rabbit anti-TH (Millipore, AB152, RRID:AB_390204, 1:1000) or rabbit anti-αSyn [Cell Signaling Technology, 4179 (D37A6), RRID:AB_1904156, 1:1000] in 10% GS/TBT at 4°C in a humidity chamber. Slides were then washed 2 × 15 min in TBT, and 1 × 15min in TBS. Slides were then incubated with 0.3% hydrogen peroxide (Sigma, H1009)/TBS to quench endogenous peroxidases for 15 min. Slides were then rinsed in tap water, before incubation with a goat anti-rabbit IgG HRP-conjugated antibody (Sigma, A6154, RRID:AB_258284, 1:2000) for 30 min. Slides were then washed 2 × 15 min in TBT, and 1 × 15 min in TBS, followed by incubation with the ImmPACT^TM^ DAB substrate (Vector Labs, SK-4105), for 5–10 min. Tissue sections were imaged on the Axioscan Z.1 (Zeiss) slide scanner, using a Plan-Apochromat 20×/NA 0.8 Objective. Light source intensity was set at 221% and pixel size was 0.22 μm × 0.22 μm. Adaptive focus was set using the Onion Skin 53 strategy. Images were captured on the Hitachi HV-F202SCL with a 200 μs exposure time.

Striatal tyrosine hydroxylase images were analysed in QuPath v0.4.^27^ Briefly, a region of interest was placed around the striatum and cortex, the OD of DAB staining in the cortex was considered background and subtracted from the staining in the striatum. The OD across the A-P axis of the striatum was averaged for each animal. The staining was normalised to control animals and compared across genotypes. Nigral tyrosine hydroxylase images were analysed using the cell counter to count TH + cell bodies in the substantia nigra. The cell counts across the A-P axis of the nigra were averaged for each animal. Counts were normalised to control animals and compared across genotypes.

DAB immunohistochemistry for pSer129-αSyn was done at 6 weeks with the same manual protocol. At 6 months, the sections were processed on the Leica Bond Max. These brains were embedded in paraffin and sectioned. The paraffin sections were de-waxed and re-hydrated using xylene and graded alcohols. Antigen retrieval was done using Epitope retrieval buffer 1 (pH 6; Leica biosystems) for 20 min. Rabbit pS129-αSyn primary antibody (Abcam, ab51253, RRID:AB_869973, 1:500) was used. Bond wash with TBS buffer (Fischer scientific) and Tween 20 was used for washing. Briefly, the protocol was: wash 10 min, peroxide block 5 min, wash, primary antibody applied for 60 min, wash, polymer 15 min, bond wash and wash with distilled water, DAB immunostain applied for 10 min, wash with distilled water, Haematoxylin stain 5 min and final wash. Reagents were from Leica Bond Polymer Refine detection kit (DS 9800). DAB images of αSyn PFF-injected brains were scanned with Axioscanner (Zeiss) using a 20× objective NA 0.8 Plan Apochromat. QuPath open source software was used to quantify pSer126-αSyn pathology using a custom script.^27^

### Fluorescent immunohistochemistry

Tissue was prepared as above, but 10 μm sections were taken instead of 30 μm. Slides were brought up to room temperature, and then microwaved for 20 s in TBS to remove OCT. Slides were then blocked in 10% goat serum in TBS with 0.1% Triton (10% GS/TBT) for 1 h. Slides were then incubated overnight in rabbit anti-αSyn (Cell Signaling Technology, 4179 (D37A6), RRID:AB_1904156, 1:1000) and mouse IgG1 anti-synaptophysin (Abcam, ab8049, RRID:AB_2198854, 1:50) in 10% GS/TBT at 4°C in a humidity chamber. Slides were then washed 3 × 15 min in TBT. Slides were then incubated with goat anti-rabbit IgG Alexa Fluor 488 and goat anti-mouse IgG Alexa Fluor 568 [both ThermoFisher, A11008 (RRID:AB_143165), A11004 (RRID:AB_2534072), 1:1000] in 10% GS/TBT for 2 h at room temperature. Slides were then washed 3 × 15 min in TBT, followed by 5-min incubation with DAPI/TBS (1:10000), and 3 × 5 min TBS washes. Slides were then mounted using ProLong^TM^ Diamond Antifade Mountant (Thermo Fisher, P36965) and 1.5 high precision coverslips (Marienfeld, 0107172).

### Confocal microscopy and image analysis

Images were acquired on a Leica TCS SP8 5D confocal microscope. Images were taken at 63× magnification using a HC PL APO 63×/1.4 oil objective, with a refraction index of 1.518 2× Zoom. Pinhole was set to 1 AU. Images were acquired at a speed of 600 hz. Line Accumulation was set at 14. Line Sequential scanning was performed and for DAPI, the 405 nm laser was set at 2.9%. HyD detector was used to capture the emitted photons in counting mode, and gain was set at 75. For αSyn immunofluorescence detection the 488 nm laser was set at 2.99%. HyD detector was used to capture the emitted photons in counting mode, and gain was set at 70. For synaptophysin immunofluorescence detection the 594 nm laser was set at 20%. HyD detector was used to capture the emitted photons in counting mode, and gain was set at 89. Images were deconvolved with the Huygens (SVI) software using an CMLE algorithm, with 40 iterations and a signal-to-noise ratio set to 10. Deconvolved images were subsequently used for co-localisation analysis. The built-in Huygens (SVI) (pixel-based) co-localisation analyser was used to calculate Spearman’s correlation coefficient (*r*) values, and background estimation set to ‘optimised’.

### Cortical protein preparation and synaptosome isolation

Brains were harvested from twelve 6-month-old rats [4 rats (3 male and 1 female) for each genotype *Snca^+/+^*, *Snca^G51D/+^* and *Snca^G51D/G51D^*]. The frontal cortex was dissected from the rest of the brain and then bisected along the midline. Total protein was isolated from one half of the cortex and synaptosomes were isolated from the other half as previously described.^28^ Briefly, each half cortex was frozen in low-bind 2-mL Eppendorf tubes (Eppendorf, 0030108132) prior to addition of 500 μL of 0.32 M Sucrose Solution [0.32 M sucrose (SLS, CHE3650), 1 mM EDTA (Invitrogen, 15575–038), 5 mM Tris (Roche, 107089176009), pH 7.4]. Each sample was homogenised until smooth and centrifuged at 900 g for 10 min at 4°C. Supernatants were transferred to new Eppendorf tubes and each pellet was resuspended in 500 μL 0.32 M Sucrose Solution. Each sample was spun at 900 g for 10 min at 4°C, and this supernatant was combined with supernatant from first spin. The pellet from the second spin was considered the non-synaptic fraction. The combined supernatant samples were spun at 20 000 g for 20 min at 4°C, supernatant was discarded and the pellets were the synaptosome fractions. See Supplementary Fig. 1 for overview. All samples were stored at −80°C until protein extraction.

### Western blotting

Protein concentrations were determined using the Micro BCA Protein Assay Kit (Thermo Scientific, 232350), following the manufacturer’s instructions. Protein (15 μg) was incubated with 5 μL NuPAGE® Loading Buffer (4×, Life Technologies, NP0008), 2 μL 1 M DTT and made up to 20 μL with deionised water at 100°C for 5 min. The gel (4–12% Bis-Tris NuPAGE® gel, Life Technologies, NP0322BOX) was loaded into the tank with running buffer (20× NuPAGE® MOPS SDS Running Buffer in deionised water, Life Technologies, NP0001). Protein samples were loaded along with the SeeBlue Plus 2 protein marker (LC5925, Life Technologies). The gel was run at 60 V for 20 min, and then at 120 V until the blue loading dye reached the base of the gel (usually 90 min) at room temperature. Gels were removed from case and then processed for Coomassie staining (see below) or transferred for immunostaining.

PDVF membranes (Amersham Hybond ECL, GE Healthcare Life Science, RPN68D) were activated in methanol for 30 s and then incubated with transfer buffer [3.02 g Tris (Roche, 107089176009), 14.4 g glycine (Sigma, G7126), 800 mL water and 200 mL methanol (Fisher, M/3900/17)]. The gel was removed and prepared for transfer to PDVF membrane. It was then loaded into the transfer tank, transfer buffer was added and the blot was run at 300 mA for 75 min at 4°C.

The membrane was removed dried for 30 min, then reactivated in methanol (30 s). Membrane was then incubated in blocking solution (5% milk in 0.01% TBT) for 2 h at RT. After removal of blocking solution, the membrane was incubated with primary antibodies in blocking solution over-night at 4°C. Primary antibodies included (i) rabbit anti-αSyn [Cell Signaling Technology, 4179 (D37A6), RRID:AB_1904156, 1:1000], (ii) rabbit anti-Rab3b [Abcam, ab177949 (EPR12987), 1:1000], (iii) mouse IgG1 anti-β-actin-HRP (Abcam, ab20272, RRID:AB_445482, 1:1000), and (iv) rabbit anti-histone H2AX (Abcam, ab20669, RRID:AB_445689, 1:1000). The membrane was washed 3 times for 15 min in 0.01%TBT and then incubated with either goat anti-mouse IgG-HRP secondary antibody (Sigma, A4416, RRID:AB_258167, 1:2000) or goat anti-rabbit IgG-HRP secondary antibody (Sigma, A6154, RRID:AB_258284, 1:2000) in blocking buffer for 1 h at room temperature. Subsequently, the membrane was washed in 0.01%TBT 3 times for 15 min before HRP was detected using the Pierce ECL Western Blot substrate kit (Thermo Scientific, 32109), according to manufacturer’s instructions. Membrane was placed in an acetate cassette and then imaged on the LI-COR Odyssey® Fc, using the 700 nm channel (30 s exposure) for ladder, and Chemi channel (10-min exposure) for antibody of interest. LI-COR Image Studio^TM^ was used to analyse images, box (size maintained between samples) was placed around band and OD was measured within box. This was then normalised to loading control OD (analysed in same way). Samples were expressed as % of control samples.

### Coomassie staining and pooling samples

Following gel run, gel was removed from case and incubated in Coomassie stain [50%v/v Methanol (Fisher, M/3900/17), 10%v/v acetic acid (SLS, CHE1014), 0.2% Coomassie Brilliant blue R-250 (Sigma, B7920)] for 30 min. Gels were then de-stained by 4 × 1 h washes in De-stain solution (7.5%v/v methanol, 10%v/v glacial acetic acid). Gels were then placed in an acetate cassette and imaged on the LI-COR Odyssey® Fc, using the 700 nm channel and exposed for 10 min. Using LI-COR Image StudioTM, OD of staining was determined for each sample at 4 weights, full lane 28–62 kDa, 62–198 kDa and 2–28 kDa. Following this, samples for tandem mass tag (TMT)-Mass spectrometry were pooled, and 50 μg of each sample was added to respective pool and made up to 100 μL with label free buffer. Coomassie gels were run again and analysed by the same method to check equivalency of loading.

### Quantitative mass spectrometry

Samples for proteomics were performed by the Fingerprints Facility, University of Dundee. Sample preparation and protein identification and quantification analysis by mass spectrometry were carried out as previously described.^28^ MaxQuant Version 1.6.0.16 was used for the assignment of peptides to protein using the uniport-rat-jan2018.fasta file. A min and max peptide length of 8 and 25 a respectively was used for unspecified search. The mass spectrometry proteomics data have been deposited to the ProteomeXchange Consortium via the PRIDE partner repository^29^ with the data set identifier PXD044776.

### Pairwise comparisons and pathway analysis

Following filtering to remove non-unique and unquantified peptides, pairwise comparisons were made between the different mutant samples and *Snca^+/+^* for both the cortical and synaptosome samples. In all cases Log2(Fold Change) compared with *Snca^+/+^* was ranked in order (Supplementary Table 1), and proteins were taken forward for pathway analysis if their expression was changed 20% up [Log2(FC) > 0.26] or down [Log2(FC)<−0.32] relative to *Snca^+/+^*. Protein lists generated by pairwise analysis were uploaded to g:profiler (http://biit.cs.ut.ee/gprofiler/gost), an online tool which performs functional enrichment analysis, mapping genes to known functional information sources and detects statistically significantly enriched pathways.^30^ Organism was set to *Rattus norvegicus* and significance threshold were set at 0.05 based on the g:SCS threshold algorithm. We identified enriched biological pathways using the Kyoto Encyclopaedia of Genes and Genomes (KEGG)^31^ (Supplementary Table 2).

### Stereotactic injection of human αSyn^WT^ PFFs

Human αSyn protein was produced in bacteria as previously described.^32^ Briefly, recombinant αSyn protein, expressed from the pRK172-hSNCA construct in *E. coli* B21 (DE3) cells, was purified by gel filtration and ion exchange chromatography. Purified αSyn was diluted to 360 mM (5 mg/mL) in sterile Dulbecco’s PBS prior to agitation (1000 rpm at 37°C) for 7 days in an Eppendorf Thermomixer C to form fibrils as previously described.^33^ PFFs were produced from αSyn fibrils by sonication in a Bioruptor Pico (Diagenode) at high power for 10 cycles (30 s on, 30 s off at 10°C). The same batch of αSyn fibrils were used from all rats, and the sonication to produce PFFs was performed on the day of injection for each cohort. Rats were anaesthetised using isoflurane per standard protocol. Depth of anaesthesia checked using tail pinch and blink reflex. Each rat was positioned on the stereotaxy frame (Stoelting, 51950), hair shaved, scalp skin cleaned with iodine and 2% Lidocaine administered subcutaneously. A clear drape was applied. Lacrilube to both eyes. Under sterile conditions, a vertical midline scalp incision (1 cm) was performed and skin retracted using retractors (Stoelting, 52124). Bregma was located using the digital arm manipulator and marked with a shallow burr hole (Stoelting, 51449 V). Target co-ordinates (AP 3.2 ML −1.5 DV −2.5) were used to place burr hole. A 10 µL Hamilton syringe (Stoelting, 51105), pre-filled with human wild-type human αSyn PFFs (10 µg in 4 µL),^34^ was used to penetrate the dura and to reach the target co-ordinates for injection. The αSyn PFFs were injected at 1 µL/min rate with the Quintessential Stereotaxic Injector and the needle left in situ for 2 min. This was done to allow αSyn PFFs to settle locally and the needle slowly retracted to minimise reflux. The skin was sutured with dissolvable 4–0 Vicryl sutures. Post-operatively, rats were placed on a heat pad at 30°C (Stoelting, 53850-RR) until motor recovery in a clean cage. The wound was checked and rats weighed regularly. Mash food and analgesia in drinking water (Rimadyl, 5 mg/kg) and Vetergesic jelly (0.5 mg/kg) were given in the post-operative period.

### Statistical analysis

The Shapiro-Wilk test was used to determine if data fell into a normal distribution and the Levene’s test for homoscedasticity was used to determine if variances were equal. For normally distributed data, Graph Pad Prism v.6.0c was used to generate a parametric one-way ANOVAs with Tukey’s multiple comparisons test to determine statistical significance of differences between *Snca^+/+^*, *Snca^G51D/+^* and *Snca^G51D/G51D^* animals. In the case of analysis just comparing *Snca^+/+^* and *Snca^G51D/+^* or *Snca^+/+^* and *Snca^G51D/G51D^* then unpaired two-tailed Student’s *t*-test was used. If data was not normally distributed, a non-parametric Kruskal-Wallis test with Dunn’s multiple comparisons test was performed to determine statistical significance of differences between *Snca^+/+^*, *Snca^G51D/+^* and *Snca^G51D/G51D^* animals. In the case of analysis just comparing *Snca^+/+^* and *Snca^G51D/+^* or *Snca^+/+^* and *Snca^G51D/G51D^* animals, then a non-parametric unpaired two-tailed Mann–Whitney test was used. See individual figure legends for details. *P* < 0.05 was considered significant.

## Results

### G51D mutagenesis of *Snca* does not significantly alter the overall abundance of αSyn protein

**Figure 1:**
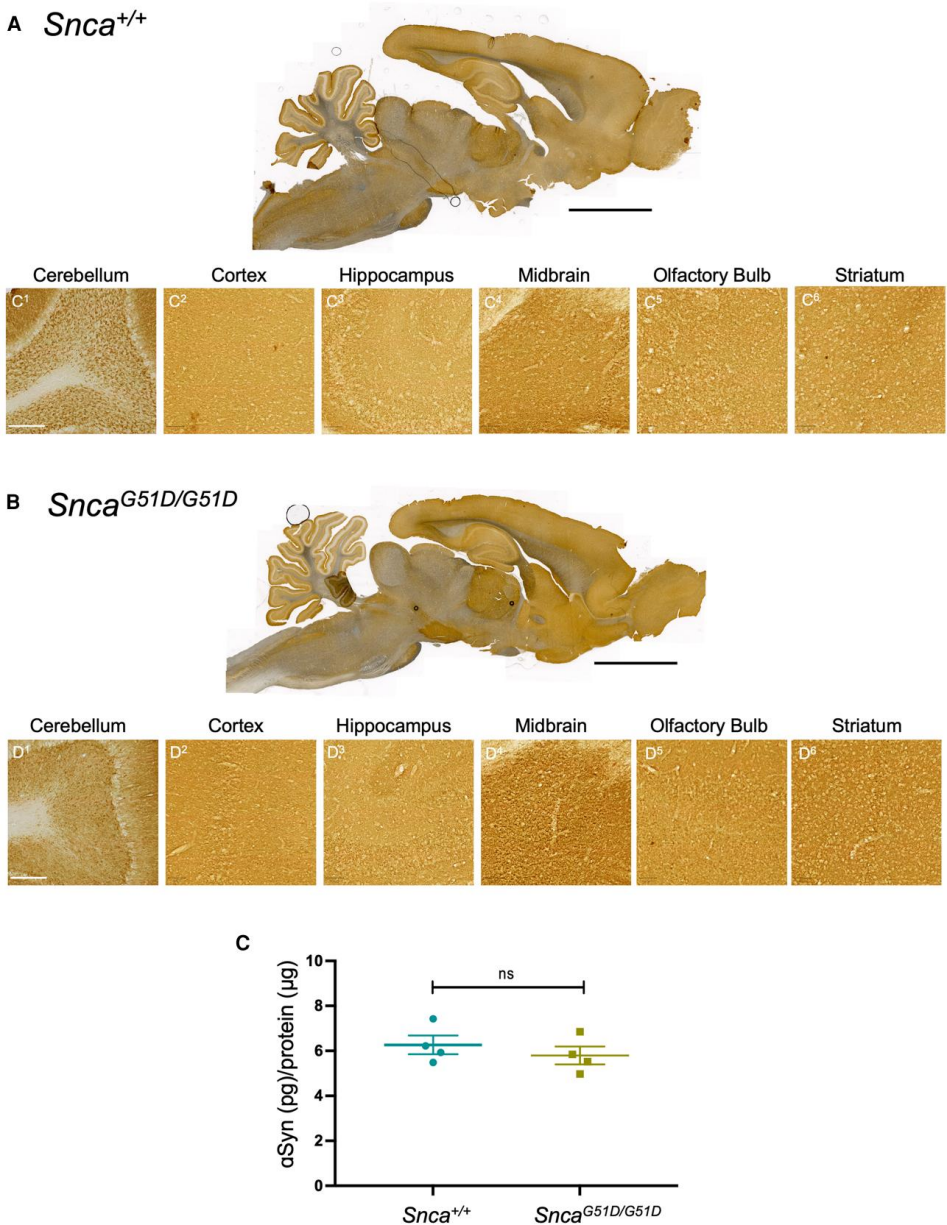
**αSyn protein levels are not significantly altered by the G51D mutation.** (**A**, **B**) DAB immunohistochemistry for αSyn in sagittal sections of 12-month-old wild-type and *Snca^G51D/G51D^* brains. Scale bar, 5 mm. There are no differences in αSyn levels between genotypes and no inclusion formation in the *Snca^G51D/G51D^* brains. C^1-6^, D^1-6^: High magnification of different brain regions. Scale bar, 200 μm. (**C**) ELISA measuring αSyn concentration in the cortex relative to total protein. There is no difference (*P* = 0.44, unpaired two-tailed *t*-test) in αSyn abundance in the cortex of *Snca^G51D/G51D^* (5.78 ± 0.79 pg/μg) compared with controls (6.27 ± 0.83 pg/μg) at 12 months of age, *N* = 4 (2 males, 2 females) animals per genotype. ns, not significant. Data presented as mean ± SEM.

The introduction of a GGA-to-GAT (G51D) codon change into the endogenous rat *Snca* gene did not affect embryonic or fetal development, and progeny from heterozygous matings were born in Mendelian ratios.^16^ We first explored whether the G51D mutation impacted on the level of αSyn expression across different brain regions. DAB immunohistochemistry for total αSyn showed similar total and regional expression in *Snca^+/+^* and *Snca^G51D/G51D^* rat brains across multiple regions in both male and female rats (Fig. 1A and B). Further, total αSyn protein levels, measured using ELISA, from the cortex of the *Snca^+/+^* and *Snca^G51D/G51D^* rats at 12 months were not significantly different (Fig. 1C). RT-qPCR was performed using RNA extracted from the brainstem of *Snca^+/+^* and *Snca^G51D/G51D^* rats and no significant change in *Snca* mRNA levels was observed at 12 months (Supplementary Fig. 2). The comparable appearance of αSyn immunostaining between genotypes, in each of the brain regions investigated, suggests that the G51D mutant in *Snca* does not significantly alter the abundance of αSyn protein.

### αSyn^G51D^ is mis-localised from the synapse in *Snca^G51D/G51D^* rat brain

**Figure 2:**
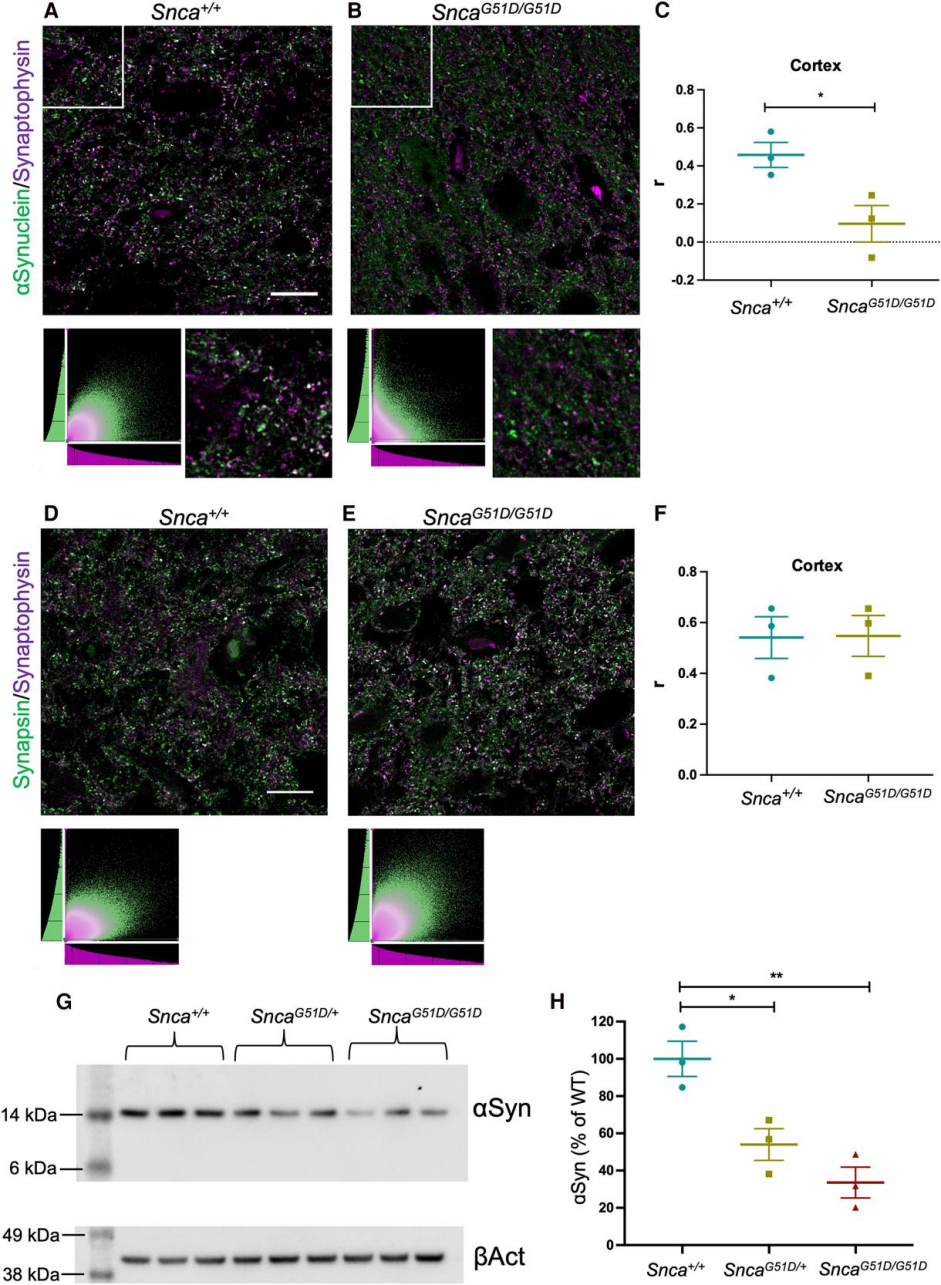
**αSyn is depleted from synapses in *Snca^G51D/G51D^* rat cortex.** (**A**, **B**) Representative deconvolved confocal images showing co-localisation between αSyn and synaptophysin, a pre-synaptic marker for *Snca^+/+^* and *Snca^G51D/G51D^* rat cortex at 6 months of age. Below confocal image is co-localisation plot showing pixel intensity in green channel (*y*-axis), plotted against pixel intensity in magenta channel (*x*-axis) and inset images are zoom of white boxes. Scale bar, 15 μm. **(C)** Comparison of object Spearman’s (*r*) values for different genotypes. There was a significant decrease in the average *r* value between *Snca^+/+^* and *Snca^G51D/G51D^* in the cortex (*N* = 3 per genotype, *Snca^+/+^*=0.458 ± 0.11, *Snca^G51D/G51D^* = 0.095 ± 0.17, *P* = 0.035, unpaired two-tailed *t*-test). (**D**, **E**) Representative deconvolved confocal image showing co-localisation between synapsin and synaptophysin, two pre-synaptic markers. Below confocal image is co-localisation plot showing pixel intensity in green channel (*y*-axis), plotted against pixel intensity in magenta channel (*x*-axis) D = *Snca^+/+^* and E = *Snca^G51D/G51D^*. (**F**) Comparison of object Spearman’s (*r*) values for *Snca^+/+^* (*N* = 3) and *Snca^G51D/G51D^* (*N* = 3). There was no difference between the average *r* value between *Snca^+/+^* (0.54 ± 0.14) and *Snca^G51D/G51D^* (0.58 ± 0.14) suggesting that synaptic structure is maintained in the *Snca^G51D/G51D^* animals. Data presented as mean ± SEM, *N* = 3 (2 males, 1 female) animals per genotype. Scale bar, 15 μm. (**G**) Western blot for αSyn in synaptosome samples of all genotypes of 6-month-old rats, with β-Actin (βAct) loading control. Uncropped western blots can be found in Supplementary Fig. 4A. (**H**) Quantification of αSyn levels in **G**, relative to βAct and normalized to *Snca^+/+^*. There is a significant effect of genotype (*P* = 0.0045, one-way ANOVA) on the levels of αSyn in the cortical synaptosomes of *Snca^+/+^*, *Snca^G51D/+^* (54.0%±14.5) and *Snca^G51D/G51D^* (33.6%±14.3) rats at 6 months and multiple comparison analysis showed significant differences between *Snca^+/+^*and *Snca^G51D/+^* (*P* < 0.05) and *Snca^+/+^* and *Snca^G51D/G51D^* (*P* < 0.01), *N* = 3 (2 males and 1 female).

Given that abundance of αSyn protein was not significantly changed in *Snca^G51D/G51D^* rats, we next examined whether there was any change in the subcellular localisation of αSyn. Thus, sections of cortex from *Snca^+/+^* and *Snca^G51D/G51D^* rats were immunostained for αSyn and the pre-synaptic marker, synaptophysin. Image analysis of αSyn and synaptophysin localisation in the cortex indicated that there was a significant decrease in the amount of co-localisation between these two proteins in the *Snca^G51D/G51D^* animals compared with *Snca^+/+^* controls (Fig. 2A–C, Supplementary Fig. 3). To confirm that this was due to a change in the localisation of αSyn and not due to a disruption in synaptic structure, another pre-synaptic marker, synapsin, was co-labelled with synaptophysin. In both *Snca^+/+^* and *Snca^G51D/G51D^* rats there was a positive and similar level of co-localisation of these synaptic markers (Fig. 2D–F). Taken together, we show that *Snca*^G51D/G51D^ rats have normal localisation of the classical synaptic markers, synaptophysin and synapsin, but contain significantly reduced amounts of αSyn protein in synapses.

To further investigate the synaptic depletion of αSyn by a complementary method, we performed western blot analysis for αSyn on isolated synaptosome samples (Fig. 2G). There was a significant difference in αSyn levels between genotypes at 6 months of age. Multiple comparison analysis showed a significant reduction in αSyn between *Snca^+/+^* and *Snca^G51D/+^* (*P* < 0.05) and *Snca^+/+^*and *Snca^G51D/G51D^* (*P* < 0.01) groups (Fig. 2H).

### Proteomic analysis showed dopaminergic synapse and Parkinson’s disease pathway dysregulation

**Figure 3:**
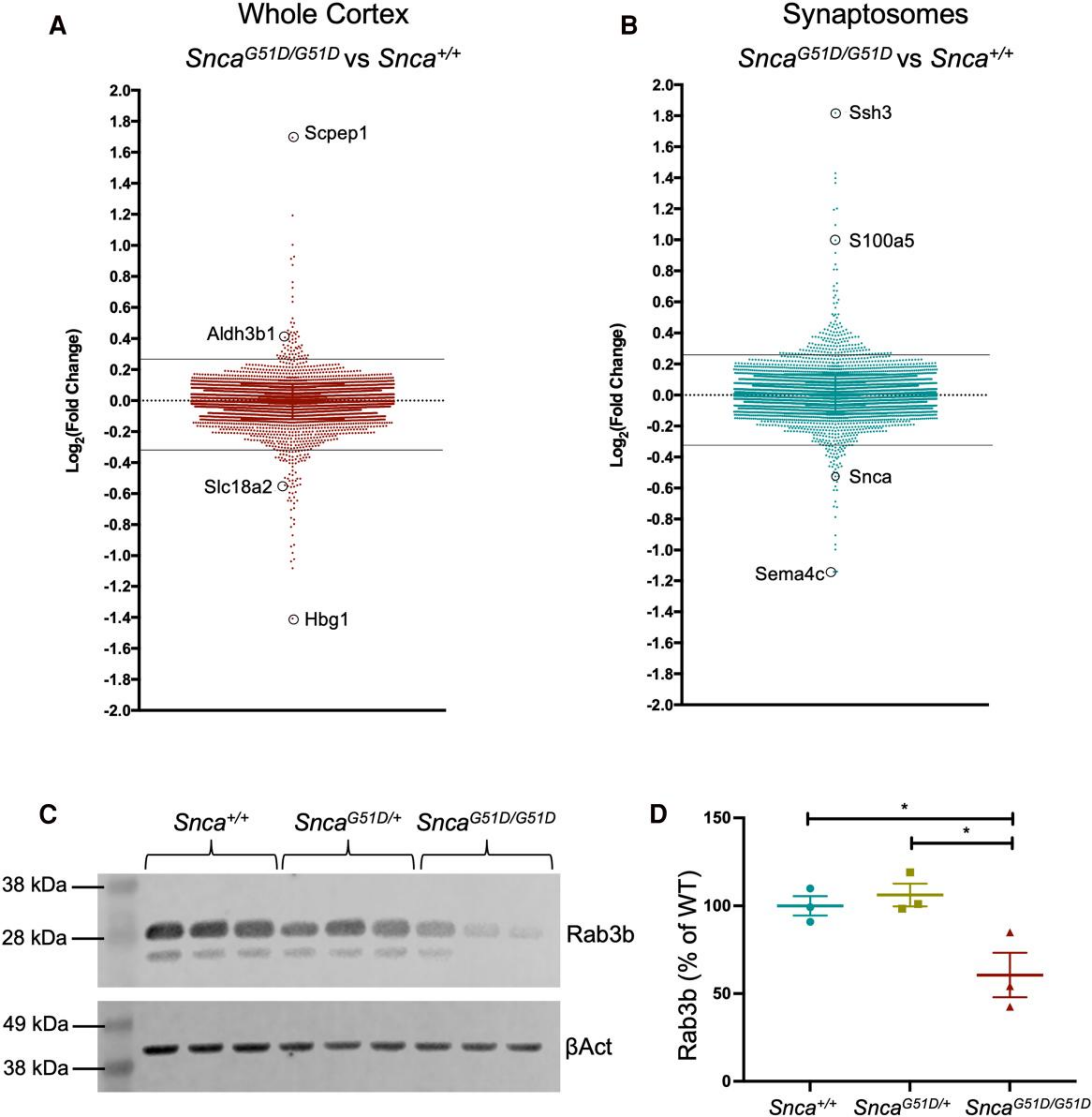
**Proteomic analysis of whole cortex and cortical synaptosomes.** (**A**) Log2(Fold Change) of all proteins in *Snca^G51D/G51D^* compared with *Snca^+/+^* cortex at 6 months of age, *N* = 4 of each genotype. Solid lines represent cut off for 20% up-regulated (>0.26) or down-regulated (<−0.32). **(B)** Log2(Fold Change) of all proteins in *Snca^G51D/G51D^* compared with *Snca^+/+^* cortical synaptosome, *N* = 4 of each genotype. See Supplementary Table 1 for lists of proteins. **(C)** Western blot for Rab3b in synaptosome samples, with β-Actin (βAct) loading control. Uncropped western blots can be found in Supplementary Fig. 4B. **(D)** Quantification of Rab3b levels in **C**, relative to βAct and normalized to *Snca^+/+^*. There is a significant effect of genotype (*P* = 0.02, one-way ANOVA) on the levels of Rab3b in the cortical synaptosomes of *Snca^+/+^*, *Snca^G51D/+^* (106.1 ± 11.2%) and *Snca^G51D/G51D^* (60.6%±21.9%) rats at 6 months and multiple comparison analysis show significant differences between *Snca^+/+^*and *Snca^G51D/G51D^* (*P* < 0.05) and *Snca^G51D/+^* and *Snca^G51D/G51D^* (*P* < 0.05). *N* = 3 (2 males and 1 female) per genotype for both analyses.

Brains were harvested from four (4) 6-month-old rats of each genotype (3 male and 1 female), and the frontal cortex was dissected from the rest of the brain and then bisected along the midline. Total protein was isolated from one half and the other half was spun through sequential sucrose gradients to isolate the synaptosomes before protein was extracted (Supplementary Fig. 1A) (see Materials and Methods for further detail). Western blotting for the H2A histone family member × (H2A.X) demonstrated the absence of the nuclear protein from the synaptic fraction (Supplementary Fig. 1B). The whole cortex and synaptosome samples of all three genotypes (*Snca^+/+^*, *Snca^G51D/+^*, *Snca^G51D/G51D^*) were processed for TMT mass spectrometry at the Fingerprints Proteomics Facility at the University of Dundee. A total of 8211 unique proteins were identified across all samples. The data set was filtered for proteins identified with at least 2 unique peptides and were observed in all six conditions, which left a total of 6640 proteins for downstream analysis. Proteins that were more than 20% up-regulated (66 proteins) or more than 20% down-regulated (79 proteins) in *Snca^G51D/G51D^* relative to *Snca^+/+^* cortex (Fig. 3A, Supplementary Table 1), were used for KEGG pathway analysis. There were 13 significantly enriched KEGG pathways for the dysregulated proteins including Parkinson’s disease (KEGG:05012), Alzheimer’s disease (KEGG:05010), serotonergic synapse (KEGG:04726), dopaminergic synapse (KEGG:04728) and oxidative phosphorylation (KEGG:00190) (Supplementary Table 2). The Parkinson’s disease and Alzheimer’s disease pathways in the *Snca^G51D/G51D^* cortex were significant due to down-regulation of proteins involved in oxidative phosphorylation (NDUFAB1, COX5A, NDUFB8), and in the case of PD dopamine transport (SLC18A2). Analysis of *Snca^G51D/+^* compared with *Snca^+/+^* cortex identified 121 up-regulated proteins and 251 down-regulated proteins (Supplementary Fig. 5, Supplementary Table 1). Pathway analysis of these protein lists identified 10 significantly enriched KEGG pathways (Supplementary Table 2), seven of which overlapped with the analysis for *Snca^G51D/G51D^* cortex, including Parkinson’s disease (KEGG:05012), serotonergic synapse (KEGG:04726), and dopaminergic synapse (KEGG:04728).

### Dysregulation of serotonergic synapse and complement pathway in *Snca^G51D^* synaptosomes

Synaptosomes were isolated from the cortex of the same 6-month-old rats (4 of each genotype) used for whole cortex analysis. A total of 163 proteins were 20% up-regulated and 60 proteins were 20% down-regulated, including αSyn, in the *Snca^G51D/G51D^* synaptosome list compared with *Snca^+/+^* (Fig. 3B, Supplementary Table 1). The comparison of *Snca^G51D/+^* synaptosome proteins to the *Snca^+/+^* list yielded 134 proteins up-regulated by 20% and 139 proteins down-regulated by 20% or more, including Rab3b (Supplementary Table 1). KEGG pathway analysis of the *Snca^G51D/G51D^* synaptosome list compared with *Snca^+/+^* identified 5 significantly enriched KEGG pathways including spliceosome (KEGG:03040), serotonergic synapse (KEGG:04726) and regulation of actin cytoskeleton (KEGG:04810) (Supplementary Table 2). Pathway analysis of the *Snca^G51D/+^* synaptosome proteins compared with *Snca^+/+^* identified 3 significantly enriched KEGG pathways including complement and coagulation cascades (KEGG:04610) and regulation of actin cytoskeleton (KEGG:04810) (Supplementary Table 2). Several proteins in the complement pathway (C2, C5, C9 and carboxypeptidase B2) were up-regulated in *Snca^G51D/G51D^* or *Snca^G51D/+^* synaptosomes. Complement proteins have been implicating to play a role in the early stages of neurodegeneration including Parkinson’s,^35,36^ and aggregated αSyn has been shown to activate the complement pathway in an experimental cell culture model.^37^ Furthermore, complement and coagulation cascades was the most significantly enriched pathway in a proteomic study of two αSyn mouse models of Parkinson’s.^38^ The serotonergic synapse pathway, significantly dysregulated in *Snca^G51D/G51D^* synaptosomes, was also present in the KEGG pathway analysis for the whole cortex data. Serotonergic innervation is also known to be affected in Parkinson’s,^39,40^ which further suggests our αSyn^G51D^ rat model is exhibiting molecular signs of early Parkinson’s.

### Rab3b is down-regulated in *Snca^G51D/G51D^* cortical synaptosomes

We chose to validate the synaptosome protein Rab3b (log_2_ (Fold Change) −0.39 in *Snca^G51D/+^* and −0.30 in *Snca^G51D/G51D^* compared with *Snca^+/+^*). Rab3b is more highly expressed in rat and human ventral tegmental area than substantia nigra, and when Rab3b is over-expressed in the rat substantia nigra it protects dopaminergic neurons in the 6-OHDA lesion model.^41^ We performed western blotting for Rab3b on synaptosomes from all three genotypes. There was a significant reduction in Rab3b in synaptosomes at 6 months in *Snca^G51D/G51D^,* but not *Snca^G51D/+^*, compared with *Snca^+/+^* rats (*P* < 0.05; Fig. 3C and D), in partial agreement with the mass spectrometry data.

### Tyrosine hydroxylase-positive neurons within the substantia nigra do not degenerate in aged *Snca^G51D/G51D^* rats

Following the finding of the loss of αSyn from the synapse, we aimed to characterise the viability of the dopaminergic system in each genotype. In Parkinson’s, αSyn aggregation occurs throughout the brain, yet dopaminergic neurons are particularly susceptible to degeneration.^42^ In familial PD caused by the αSyn G51D mutation, degeneration of the substantia nigra was extensive.^10,11^ However, quantification of TH^+^ neurons in the substantia nigra of *Snca^+/+^*, *Snca^G51D/+^* and *Snca^G51D/G51D^* animals at either 6 months or 12 months indicated there was no difference between the number of TH^+^ neurons (Supplementary Fig. 6A to H). These findings suggest that in this rat model, αSyn^G51D^ does not lead to overt dopaminergic neuron death by 12 months of age. Furthermore, quantification of the OD of TH staining in the striatum, showed that there was no difference between genotypes at 6 months (Supplementary Fig. 6I to L). This data is in agreement with our ^18^F-DOPA PET imaging of this rat model that identified an increase in dopamine turnover at 16 months of age, but not a significant decrease in total dopamine synthesis.^16^

### *Snca^G51D/G51D^* brains are more susceptible to synucleinopathy following αSyn PFF injection

**Figure 4:**
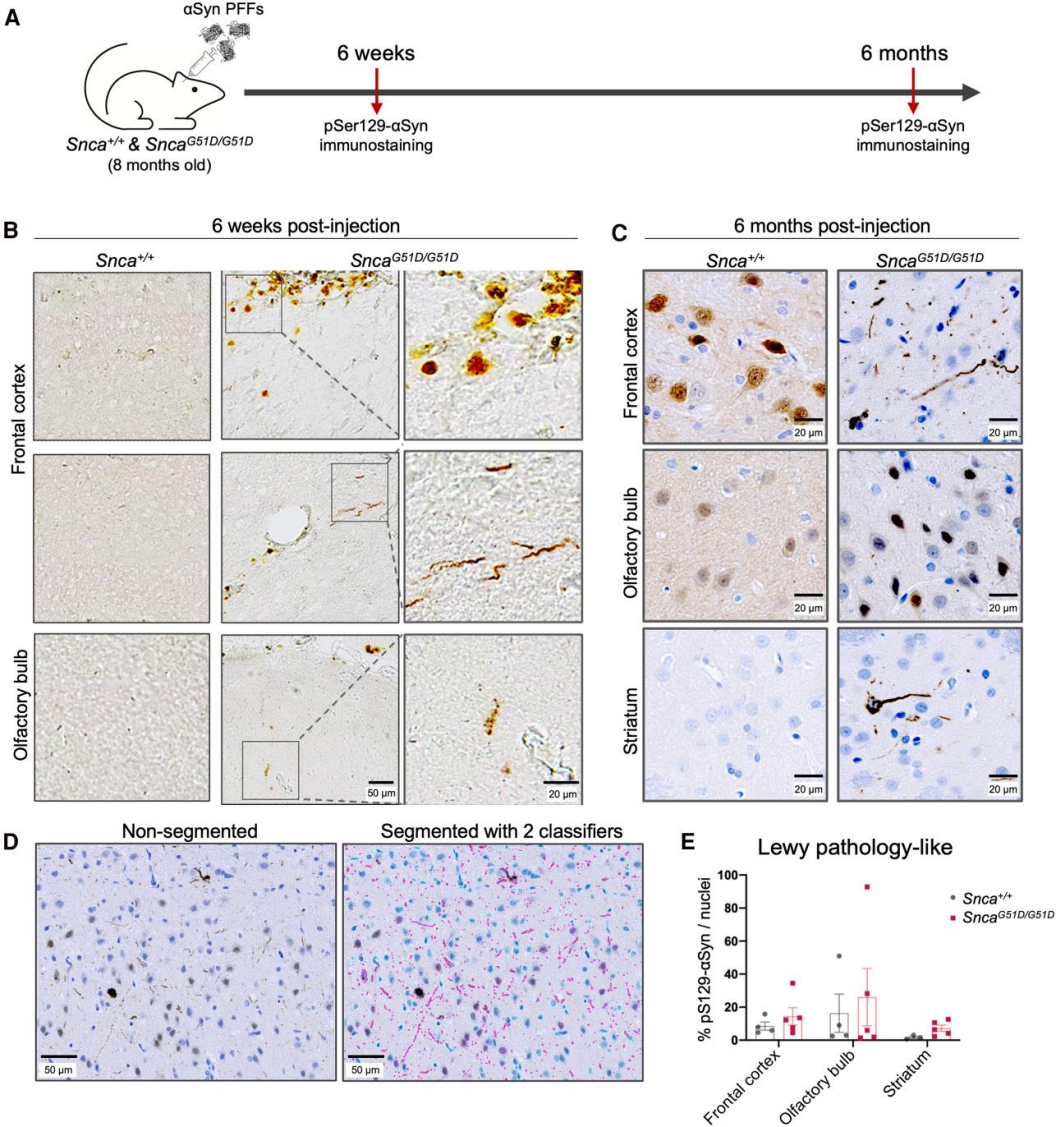
***Snca^G51D/G51D^* rats rapidly develop LP-like structures following αSyn PFF injection. (A)** Schematic of experimental time-line. *Snca^+/+^* and *Snca^G51D/G51D^* rats at 8 months of age were injected with human αSyn^WT^ PFFs into the pre-frontal cortex and processed for pSer129-αSyn DAB immunostaining at 6 weeks and 6 months post-injection. (**B**) At 6 weeks, LP-like structures formed near the injection site and the olfactory bulb in the *Snca^G51D/G51D^* rat brain, but absent from *Snca^+/+^* rat brain. Scale bar, 50 µm (20 µm, zoomed-in images). (**C**) The 6-month images include haematoxylin nuclear staining and show LP-like pathology in the olfactory bulb, frontal cortex and striatum. Scale bar, 20 µm. These inclusions were diffuse in *Snca^+/+^* compared with well-defined and intensely stained structures in *Snca^G51D/G51D^* rats. **(D)** Images to show detection of nuclei and LP-like structures for quantification, using a custom script in QuPath. **(E)** Quantification of LP-like pathology per nuclei at 6 months showed more inclusions (% of pS129-αSyn puncta per nuclei per field) in the frontal cortex, olfactory bulb and striatum of *Snca^G51D/G51D^* rats, but the differences were not statistically significant (unpaired two-tailed *t*-test; *t*(7) = −0.558, *P* = 0.59 for frontal cortex, *t*(7) = −0.137, *P* = 0.89 for olfactory bulb, *t*(6) = −2.082, *P* = 0.083 for striatum). Each data point represents an individual rat (*N* = 3 for *Snca*^+/+^ striatum, *N* = 4 for *Snca*^+/+^ frontal cortex and olfactory bulb, and *N* = 5 for all regions of *Snca*^G51D/G51D^). Data presented as mean ± SEM.

Although overt neurodegeneration was absent, proteomic data revealed that our αSyn^G51D^ rat model exhibited early molecular signs of Parkinson’s. We decided to determine whether the *Snca^G51D/G51D^* rats were more susceptible to a disease trigger mediated by αSyn PFFs. Human wild-type αSyn PFFs were introduced into *Snca^+/+^* and *Snca^G51D/G51D^* animals via intracerebral injection into the pre-frontal cortex (Fig. 4A). DAB immunostaining for pSer129-αSyn was used to detect LP-like structures. While no inclusions were seen in *Snca^+/+^* controls at 6 weeks post PFF injection, there were robust LP-like structures in the pre-frontal cortex and olfactory bulb of *Snca^G51D/G51D^* rats (Fig. 4B). At 6 months, following pre-frontal cortex αSyn PFF injection, both *Snca^+/+^* and *Snca^G51D/G51D^* rats developed LP-like inclusions, however, the overall abundance was greater at 6 months and different in appearance between the genotypes. *Snca^G51D/G51D^* homozygous mutants had well-defined inclusions that appeared to be denser and more intensely stained for pSer129-αSyn, whereas control rats had diffuse, nuclear and cytoplasmic pSer129-αSyn staining (Fig. 4C, Supplementary Fig. 7). The frontal cortex, olfactory bulb and striatum had more LP-like structures in *Snca^G51D/G51D^* rats than controls, but the differences were not statistically significant (Fig. 4D and E). These data suggest that rats expressing the αSyn^G51D^ protein in the brain are susceptible to acquiring LP-like structures more rapidly, and potentially more aggressively that rats expressing αSyn^WT^ protein.

## Discussion

This study characterises a novel rat model of α-synucleinopathy generated via CRISPR/Cas9 to introduce an endogenous *Snca^G51D^* mutation.^16^ The G51D mutation alone was insufficient to cause the formation of LP-like structures and nigrostriatal degeneration at one year. However, imaging and biochemical analyses revealed mis-localisation of αSyn from synapses in homozygous *Snca^G51D/G51D^* rats. Additionally, intracerebral injection of αSyn PFFs accelerated synucleinopathy in these mutant rats compared with the controls. Proteomic analysis uncovered molecular dysfunctions in dopaminergic and serotonergic synapses, alongside impairments in oxidative phosphorylation. Pathway analysis identified significant dysregulation in pathways associated with Parkinson’s disease and Alzheimer’s disease. This work establishes an excellent *in vivo* model to investigate αSyn-mediated disease, offering several advantages over αSyn transgenic overexpression models and promising valuable insights into early disease mechanism in α-synucleinopathies.

Unlike the *SNCA* triplication mutation, which doubles αSyn levels,^43^ we found that RNA and protein levels of total αSyn were similar in *Snca^G51D/G51D^* and control rats. This suggests that the pathogenicity of the αSyn^G51D^ protein is more aggressive than αSyn^WT^ given that the clinical phenotypic severity of both mutations in patients is similar.^42^ We expected to observe synucleinopathy in the *Snca^G51D/G51D^* rats and subsequent altered levels of total αSyn, but this was not the case at 1 year. It is possible that with even older mutant rats we may have observed this pathology. In contrast to patients with the G51D *SNCA* mutation, there was a lack of an early-onset disease phenotype in *Snca^G51D/G51D^* rats. This is further corroborated by data from Zhu and colleagues, where minipigs with *SNCA* mutations at E46K, H50Q and G51D in combination, did not lead to LP-like structures at 3 months, albeit the animals harboured heterozygous mutations and the study duration was relatively short.^15^ A recent study investigated transgenic mice expressing human *G51D-SNCA* driven by the pan-neuronal *Thy-1* promoter and they observed a modest loss of dopaminergic neurons in the substantia nigra at 12 months of age.^44^ However, this is an over-expression model and on the background of a normal level of mouse αSyn protein. A recent study by Kim *et al.*^45^ generated *Snca^G51D/G51D^* knock-in mice and observed early olfactory and gastrointestinal deficits by 6 months of age, preceding motor dysfunction, which began at 9 months.^45^ These phenotypes were accompanied by pathological αSyn phosphorylation and aggregation, suggesting a more aggressive progression compared with our rat model. In support of the lengthy times needed to observe rat phenotypes, the BAC-hSNCA transgenic rat model of αSyn overexpression did not exhibit significant formation of proteinase K insoluble αSyn deposits until 16 months of age.^17^

*In vitro* biophysical studies have shown that αSyn^G51D^ can impair phospholipid membrane affinity,^46^ and αSyn^G51D^ can form different strains of PFFs under different experimental conditions, as well as cross-seed with αSyn^WT^.^47,48^ Different pathogenic αSyn strains can cause variable phenotypes, shown using brain homogenates from PD and MSA patients that were used to trigger pathology in cells or in rodent brains, with MSA-derived fibrils being more potent disease triggers.^49^ We found αSyn^G51D^ to be mis-localised from synapses, but this was insufficient for LP-like structure formation in unperturbed animals. Fares and colleagues observed few inclusions resulting from αSyn^G51D^ but found an increase in mitochondrial fragmentation in cultured neurons.^24^ Interestingly, our proteomic analysis of *Snca^G51D/G51D^* cortex showed synaptic and oxidative phosphorylation dysfunction. It is possible that synaptic and mitochondrial dysfunction precede overt αSyn inclusion formation. The lack of LP-like inclusions may explain the lack of observed dopaminergic neuronal loss in *Snca^G51D/G51D^* rats at 1 year. Although it is important to note that αSyn is more abundant in cortical excitatory synapses than dopaminergic synapses,^50^ cortical dysfunction is more challenging to assess in a rodent models. Our data also showed spliceosome pathway dysfunction due to αSyn^G51D^ and one study has demonstrated that the components of the spliceosome are present and functional in dendrites and may provide an additional level of local translational control.^51^

Introducing a secondary disease trigger was essential to reveal underlying synucleinopathy in *Snca^G51D/G51D^* rats. While the frontal cortex is not a typical site for intracerebral αSyn injection,^52-56^ it aligns with the G51D pathology observed post-mortem and relevant to α-synucleinopathies like MSA. Further, it was useful to explore subsequent temporospatial LP-like structures in interconnected regions such as the striatum. In both genotypes, the pathology was most apparent near the injection site and structures in close proximity including the olfactory bulbs, but *Snca^G51D/G51D^* rats developed synucleinopathy more rapidly than controls. The LP-like inclusions appeared well-defined and mature in *Snca^G51D/G51D^* rats, relative to diffuse nuclear and cytoplasmic pSer129-αSyn immunostaining in wild-type rats. This suggests that the αSyn^G51D^ protein in *Snca^G51D/G51D^* rat brain is in a state that increases its propensity to form LP-like structures at a faster rate when challenged with αSyn PFFs.

Whilst αSyn deletion or overexpression does not impair neurogenesis and development of immature neurons,^57,58^ αSyn may have a role at the synapse in aged mature neurons. A limitation of this study is absence of data from rats older than one year, as well as the lack of investigation into potential gender differences. Another limitation is that we do not investigate the oligomeric state of αSyn in either of the genotypes. If synaptic and mitochondrial dysfunction are early events in the αSyn^G51D^ model, analysis of oligomeric αSyn using super-resolution microscopy may reveal pathological aggregates in these subcellular locations in *Snca^G51D/G51D^* rats.^59,60^ Furthermore, from post-mortem data we know that glia including oligodendrocytes exhibit significant LP,^10^ and this remains an area to be explored in this rodent model. Caveats regarding αSyn^WT^ PFFs as a disease trigger include the fact that the exact concentration cannot be precisely measured and delivered into the brain and different strains have different pathogenicity. Indeed Hayakawa and team shows that αSyn^G51D^ PFFs were more potent inducers of synucleinopathy than αSyn^WT^ PFFs,^61^ and thus may have shown a greater difference between then genotypes following intracerebral injection into wild-type and *Snca^G51D/G51D^* rats.

In summary, this study characterized a novel rat model of synucleinopathy, harbouring an endogenous *Snca^G51D^* mutation, expressing physiological levels of αSyn and without the limitations associated with ectopic, heterologous transgenes. The combination of synaptic and mitochondrial dysfunction and the αSyn^G51D^ mis-localisation from the synapse may be early events in the disease process, increasing the propensity for overt disease following a second disease trigger. Our disease-relevant model provided an opportunity to investigate the underlying mechanism of the *SNCA^G51D^* point mutation that exerts a severe clinical phenotype in patients. This model offers a valuable platform to uncover mechanistic insights and accelerate the development of disease-modifying treatments for α-synucleinopathies.

## Supplementary Material

fcaf224_Supplementary_Data

## Data Availability

The proteomics data is available from the ProteomeXchange (https://www.proteomexchange.org/) via the accession number: PXD044776. All other requests for data should be directed to the corresponding author: tilo.kunath@ed.ac.uk.
