# Supplementary material for: Glycine-to-aspartic acid mutation at codon 51 in Snca disrupts the synaptic localisation of α-synuclein and enhances its propensity for synucleinopathy
Source: Brain Commun. 2025 Jun 6;7(3):fcaf224. doi: 10.1093/braincomms/fcaf224 (PMC12198753; doi:10.1093/braincomms/fcaf224)
Supplement: fcaf224_Supplementary_Data [file fcaf224_supplementary_data.zip › Supplementary_material.docx]

**Glycine-to-aspartic acid mutation at codon 51 in *Snca* disrupts the synaptic localisation of α-synuclein and enhances its propensity for synucleinopathy**

Stephen West, Ammar Natalwala, Karamjit Singh Dolt, Douglas J. Lamont, Melanie McMillan, Kelvin Luk, Tomoji Mashimo, Tilo Kunath

**Supplementary Figure 1**

**
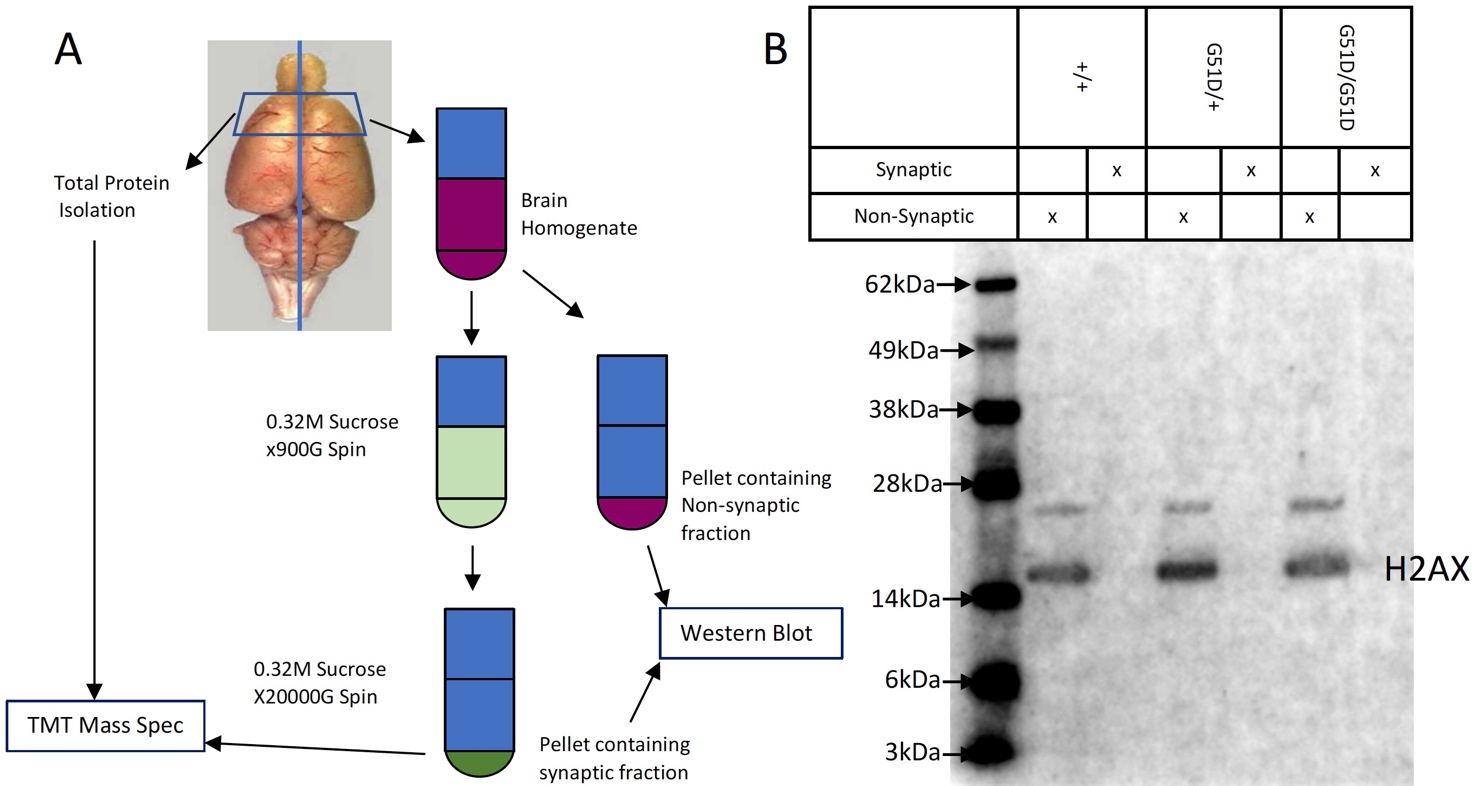
**

**Supplementary Figure 1. Whole cortex and synaptosome isolation for proteomic analysis. (A)** Diagram outlining synaptosome and whole brain protein isolation for Tandem Mass Tag mass spectrometry (TMT Mass Spec) experiments. **(B)** Western blot showing loss of nuclear protein H2AX from the synaptosome samples of all three genotypes (N = 1 per genotype).

**Supplementary Figure 2**

**
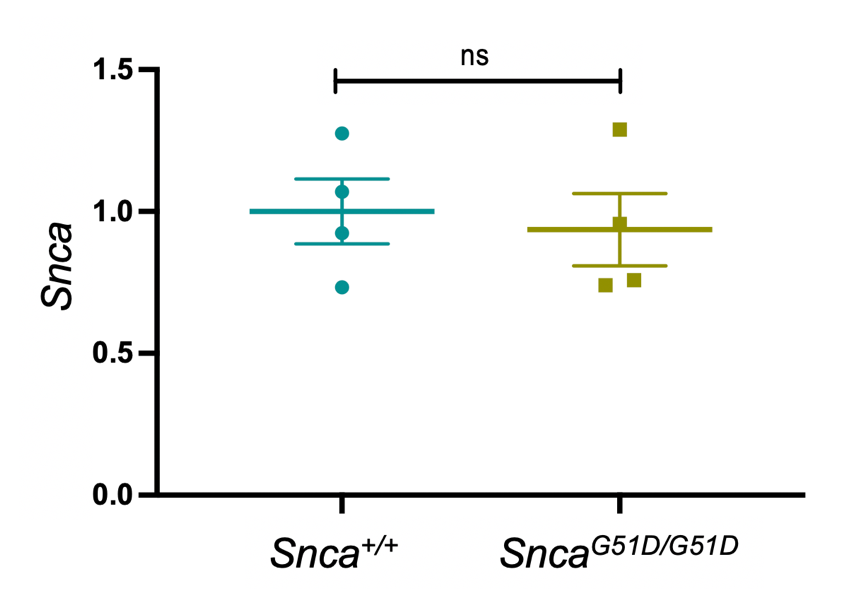
**

**Supplementary Figure 2. *Snca* transcript levels are not altered by the G51D mutation.** RT-qPCR showing *Snca* gene transcript levels from the brain stem relative to the arithmetic mean of *Hprt* and *Tbp* and normalized to controls. Each data point represents an individual rat. There is no significant difference in transcription levels of *Snca* in *Snca^+/+^* and *Snca^G51D/G51D^* 12-month-old animals, (N = 4 (2 males, 2 females) rats per genotype; *t*(6) = 0.3744, *P* = 0.72, unpaired two-tailed *t*-test). Data presented as mean±SEM.

**Supplementary Figure 3**

**
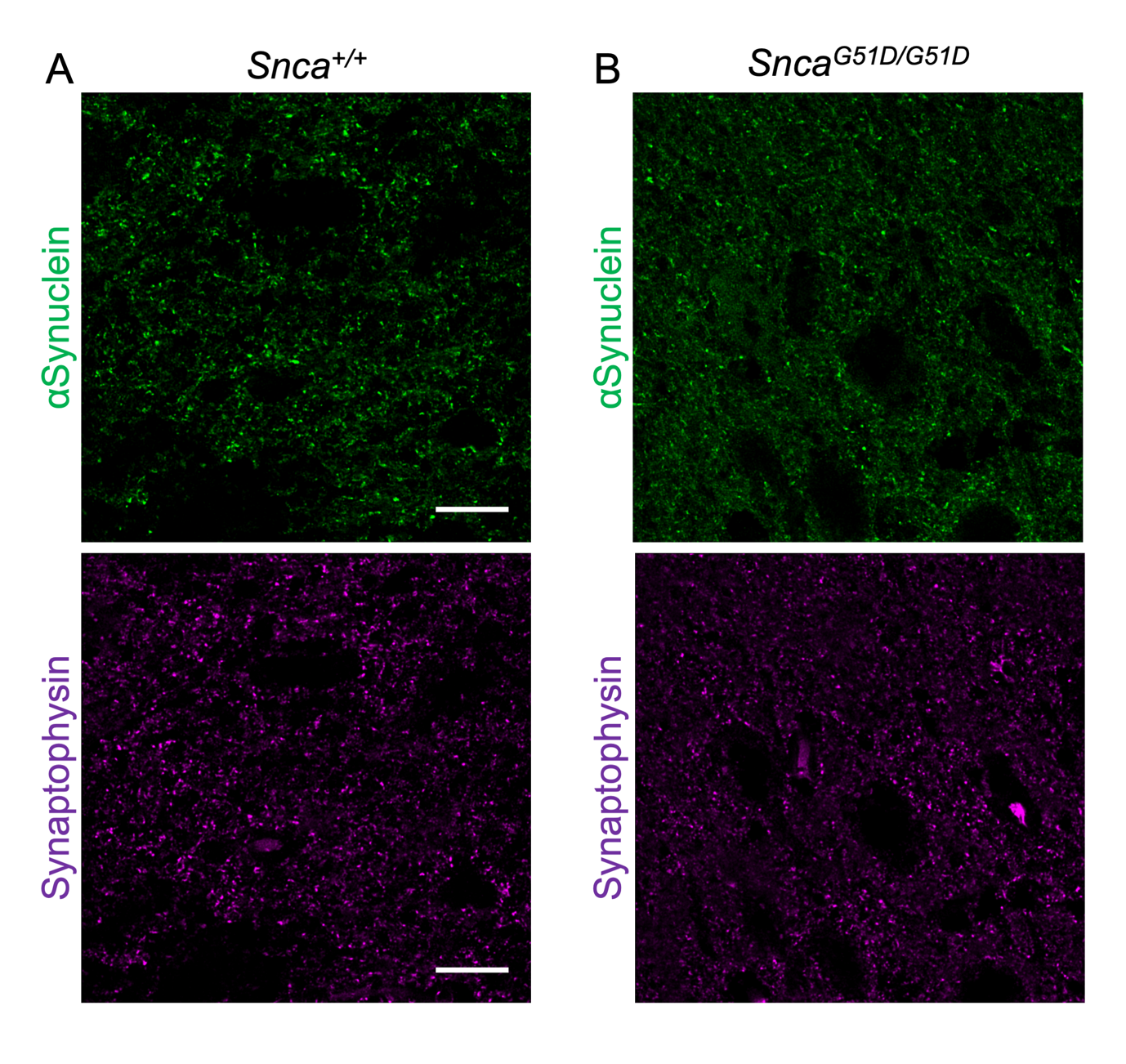
**

**Supplementary Figure 3. αSynuclein and synaptophysin of 6-month-of *Snca^+/+^* and *Snca^G51D/G51D^* rat cortex. (A, B)** Representative deconvolved confocal images of αSyn and synaptophysin immunostaining of *Snca^+/+^* and *Snca^G51D/G51D^* rat cortex at 6 months of age. Scale bar, 15 μm.

**Supplementary Figure 4**

**
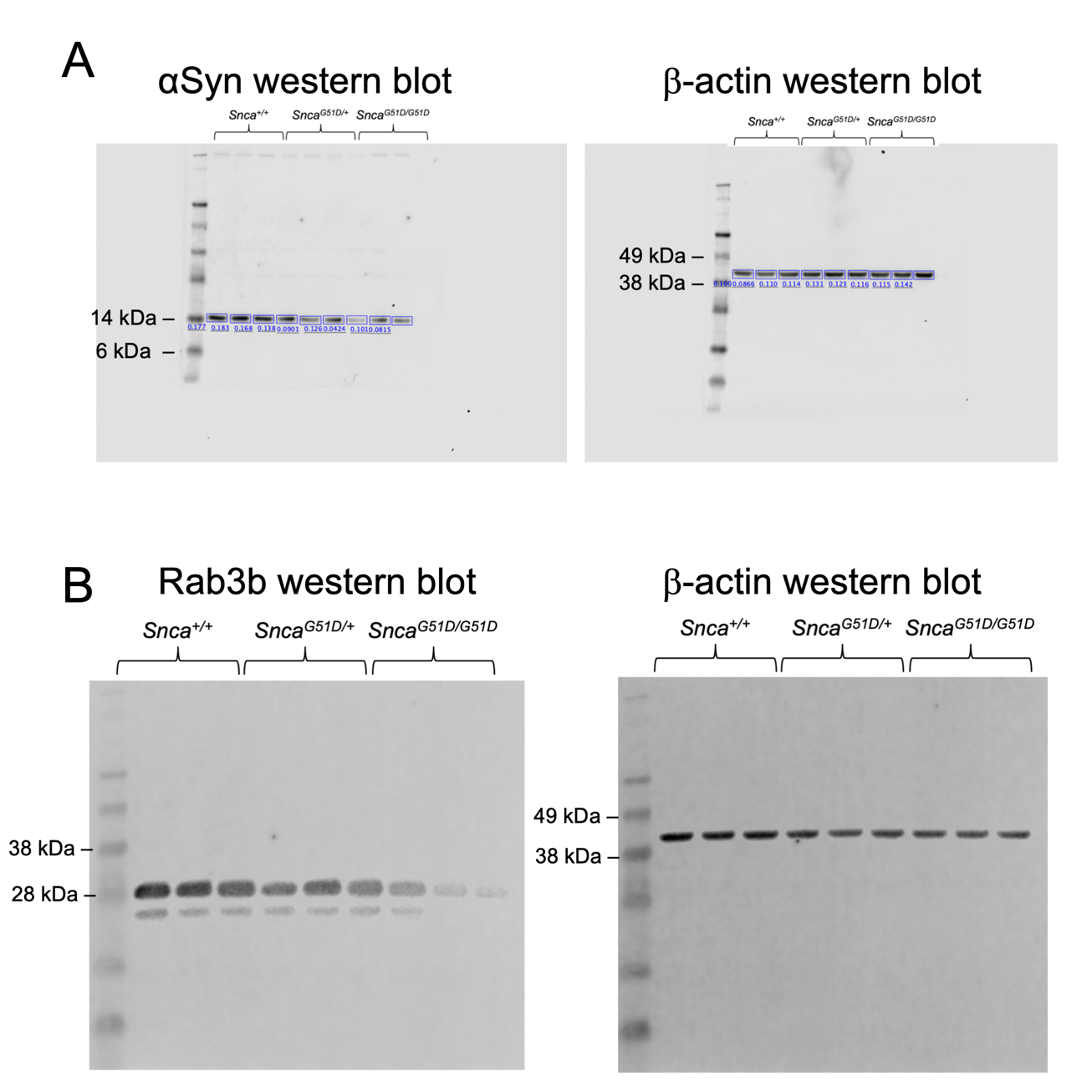
**

**Supplementary Figure 4. Uncropped western blots. (A)** Uncropped western blots related to Figure 2G. **(B)** Uncropped western blots related to Figure 3C.

**Supplementary Figure 5**

**
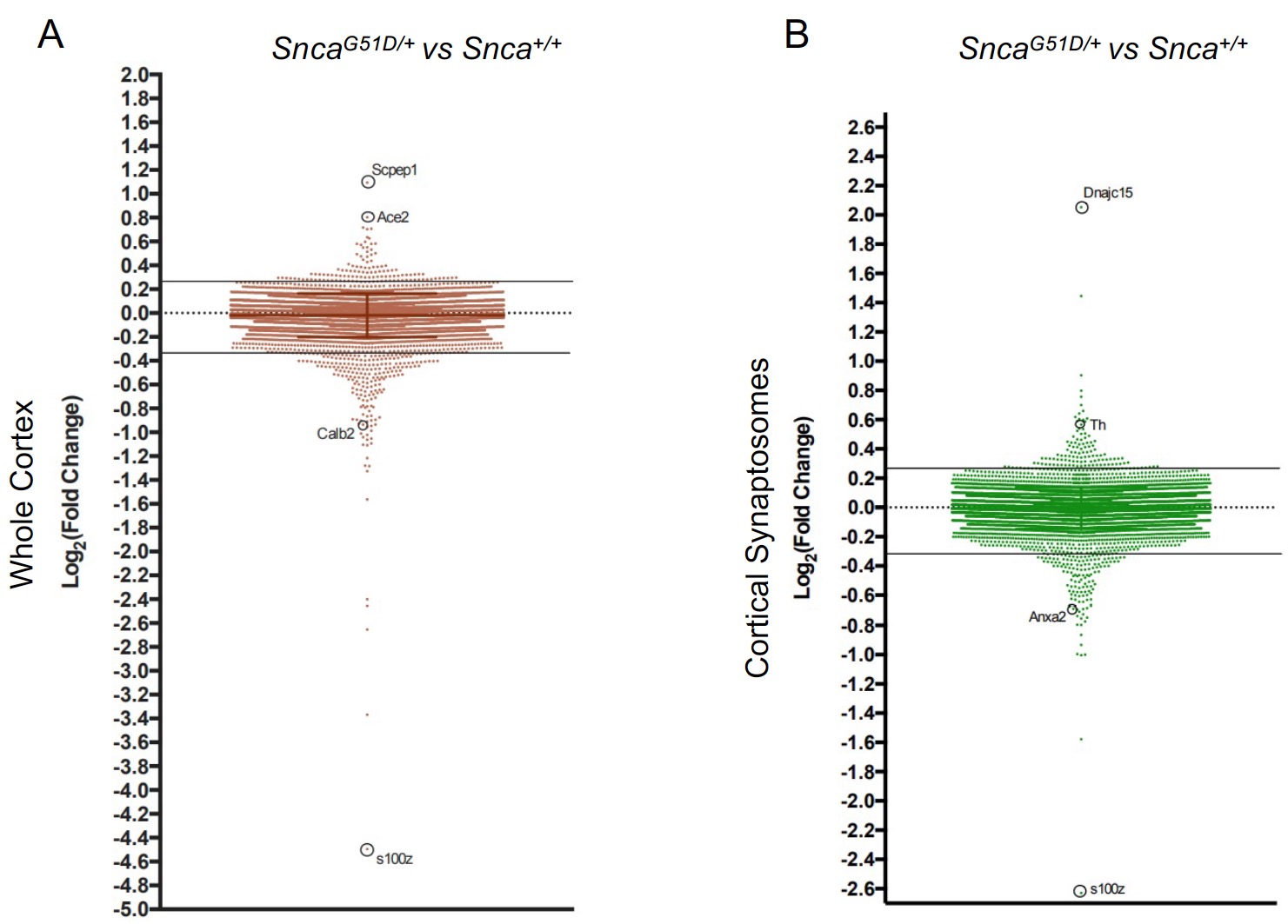
**

**Supplementary Figure 5. Proteomic analysis of *Snca^G51D/+^* whole cortex and cortical synaptosomes. (A)** Log2(Fold Change) of all proteins in *Snca^G51D/+^* compared to *Snca^+/+^* cortex. Solid lines represent cut off for 20% up-regulated (>0.26) or down-regulated (<-0.32). (121 up-regulated and 251 down-regulated proteins), N = 4 of each genotype. **(B)** Log2(Fold Change) of all proteins in *Snca^G51D/+^* compared to *Snca^+/+^* cortical synaptosome preparations (134 up-regulated and 139 down-regulated proteins), N = 4 of each genotype. See Supplementary Table 1 for full lists of proteins.

**Supplementary Figure 6**


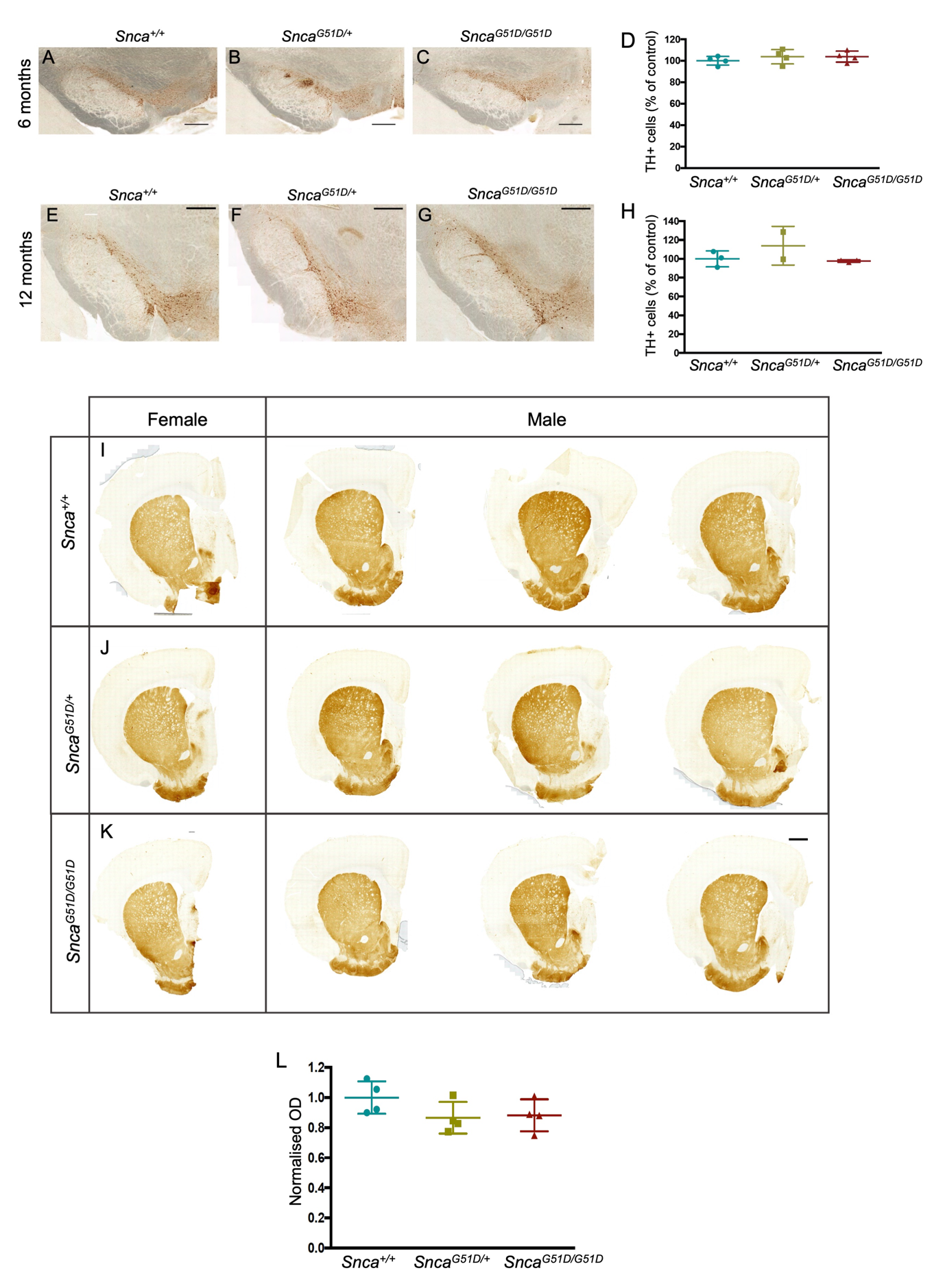


**Supplementary Figure 6. *Substantia nigra* TH+ neurons do not significantly degenerate in the *Snca^G51D/+^* or *Snca^G51D/G51D^* rats. (A-C)** Representative images of TH+ neurons in the *substantia nigra* (SN) at 6 months. Scale bar, 500 μm. **(D)** Quantification of SN TH+ neurons in different genotypes relative to controls. There was no significant difference in cell numbers between *Snca^+/+^*, *Snca^G51D/+^* and *Snca^G51D/G51D^* rats at 6 months. (N = 4 (3 males, 1 female) animals per genotype; *F*(2,9) = 0.6619, *P* = 0.54, one-way ANOVA). (**E-G)** Representative images of TH+ neurons in the SN at 12 months. Scale bar, 500 μm. (**H**) Quantification of SN TH+ neurons in different genotypes relative to controls. There was no significant difference in cell numbers between *Snca^+/+^*, *Snca^G51D/+^* and *Snca^G51D/G51D^* rats at 12 months. (N = 3 males for *Snca^+/+^* and *Snca^G51D/G51D^*, N = 2 males for *Snca^G51D/+^*; *F*(2,5) = 1.541, *P* = 0.30, one-way ANOVA). **(I-K)** Representative images of TH+ terminals in the striatum. I = *Snca^+ /+^* J = *Snca^G51D/+^* K = *Snca^G51D/G51D^*. Each coronal section is a representative slice for each animal analysed. Scale bar, 1 mm. (**L**) Quantification of striatal TH optical density (OD) in different genotypes relative to controls. There was no significant difference in OD between *Snca^+ /+^*, *Snca^G51D/+^* and *Snca^G51D/G51D^* rats at 6 months. (N = 4 (3 males, 1 female) animals per genotype; *F*(2,9) = 1.903, *P* = 0.20, one-way ANOVA). Each data point represents an individual rat and the data is presented as mean±SD.

**Supplementary Figure 7**


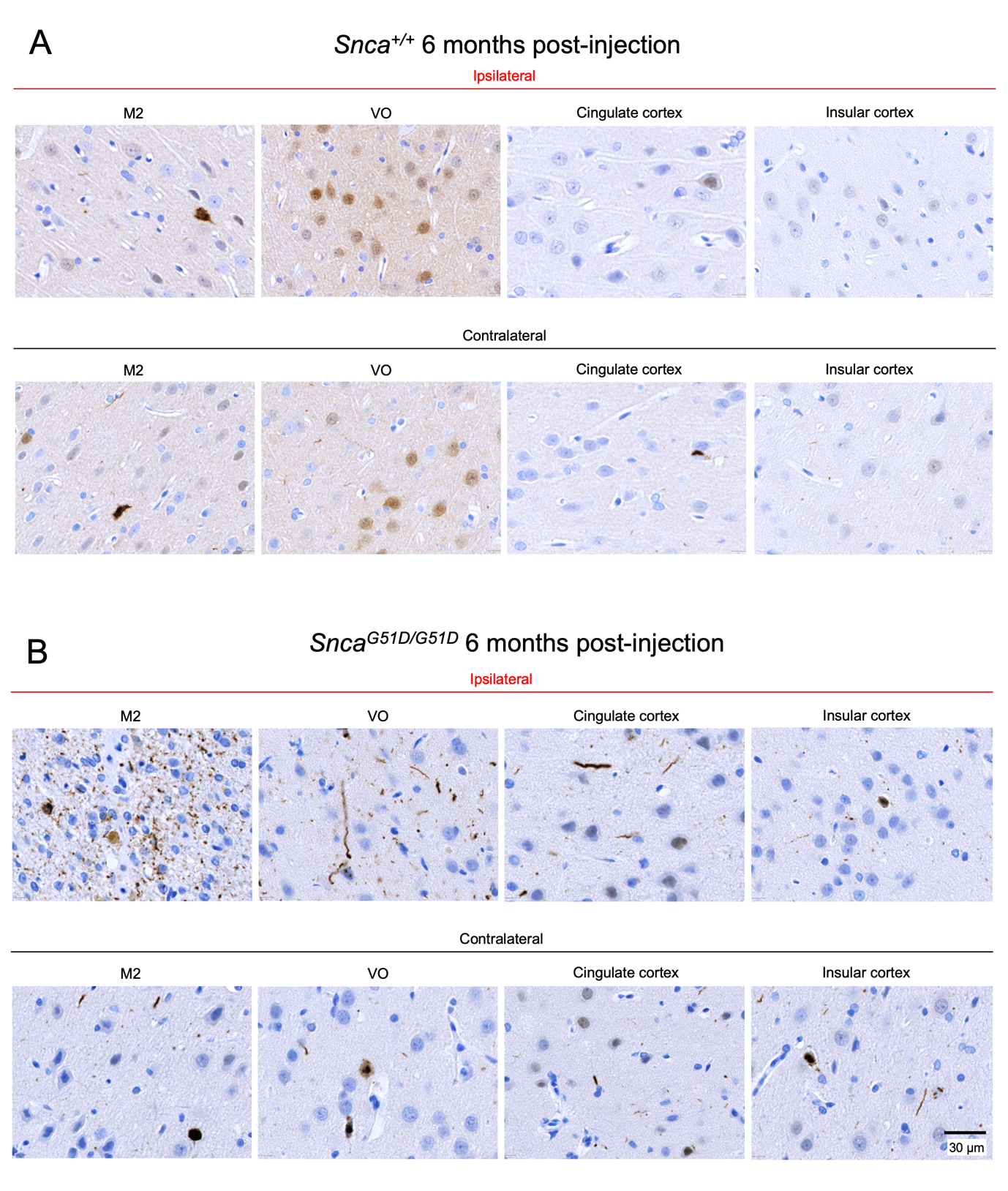


**Supplementary Figure 7. pSer129-αSyn pathology in cortical areas of PFF-injected *Snca^+/+^* and *Snca^G51D/G51D^* rats. (A,B)** pSer129-αSyn DAB immunostaining of *Snca^+/+^* and *Snca^G51D/G51D^* rat cortical regions 6 months after injection with human αSyn^WT^ PFFs into the pre-frontal cortex. Scale bar, 30 μm. M2 = secondary motor cortex, VO = ventral orbital cortex
